# A qualitative exploration of rural and semi-urban Sri Lankan men’s alcohol consumption

**DOI:** 10.1080/17441692.2019.1642366

**Published:** 2019-08-25

**Authors:** Jane Brandt Sørensen, Flemming Konradsen, Thilini Agampodi, Birgitte Refslund Sørensen, Melissa Pearson, Sisira Siribaddana, Thilde Rheinländer

**Affiliations:** a Department of Public Health, University of Copenhagen, Copenhagen, Denmark; b Faculty of Medicine, South Asian Clinical Toxicology Research Collaboration (SACTRC), University of Peradeniya, Peradeniya, Sri Lanka; c Department of Community Medicine, Faculty of Medicine and Allied Sciences, Rajarata University of Sri Lanka, Saliyapura, Sri Lanka; d Department of Anthropology, University of Copenhagen, Copenhagen, Denmark; e School of Clinical Sciences and Community Health, The University of Edinburgh, Edinburgh, Scotland; f Department of Medicine, Faculty of Medicine & Allied Sciences, Rajarata University of Sri Lanka, Saliyapura, Sri Lanka

**Keywords:** Alcohol, substance use, Bourdieu, gender, Sri Lanka

## Abstract

Harmful alcohol drinking can have health and socio-economic consequences. However, consumption is also associated with pleasure and symbolic meanings. Alcohol intake is increasing in Sri Lanka. In-depth explorations of alcohol patterns are needed to inform interventions and policies. Qualitative data were collected over 11 months in 2014 and 2015 in the North Central Province of Sri Lanka. Ten focus group discussions were conducted in gender, age and geographically (rural and semi-urban) segregated groups. Observations were conducted at alcohol selling establishments and social gatherings. Bourdieu’s concepts *practice*, *habitus, symbolic capital* and *distinction* were used for the analysis. Three groups of consumers emerged: moderate consumers, abstainers and heavy drinkers. They each exercised distinctions through social codes of conduct within and towards other groups of consumers. Symbolic capital was expressed through choice of alcohol. Norms of ‘acceptable consumption’ were defined as ‘moderate drinking’ in covert, social and contained settings. Public, uncontrolled and solitary consumption violated norms of appropriate consumption. Young consumers communicated a ‘modern lifestyle’ through their consumption. This study found that alcohol practices mirrored social norms in this Sri Lankan setting. Alcohol and drug prevention and intervention efforts should take this into account.

## Introduction

It is well-established that alcohol is a contributor to ill health and social harm (World Health Organization, 2018), including for people other than the consumer (Room et al., 2010). Globally, alcohol results in about 5.3% of all deaths and 5.1% of the burden of disease (World Health Organization, 2018). Medical and socio-economic problems stemming from alcohol drinking are also evident in Sri Lanka, where an increase in alcohol consumption has been reported. Annual per capita consumption of legal pure alcohol increased by 62% since 1998 and was estimated as 2.6 l in 2013 (Nugawela, Lewis, Szatkowski, & Langley, 2017). While this is low compared to international standards (World Health Organization, 2018), illicit alcohol consumption, mainly consumed in rural areas, was estimated to account for 40% of the total consumption between 2008 and 2010 (World Health Organization, 2014). This makes realistic figures of alcohol consumption much higher. Under-reporting of alcohol consumption is likely influenced by the main religion in Sri Lanka, Buddhism, where alcohol can be considered immoral (Gombrich & Obeyesekere, 1988). Many adults in Sri Lanka are abstainers, especially women. A cross-sectional Sri Lankan study including 4532 participants with a mean age of 46, found that 48.1% of men and only 1.2% of women reported current alcohol use (having consumed alcoholic beverages during the past 12 months) (5). Considering the widespread consumption of illicit alcohol and the large group of abstainers (65.7%) (Katulanda et al., 2014), those who consume alcohol often do so at rates above the per capita estimates.

Several policies have been implemented in Sri Lanka to decrease alcohol consumption. In 2006 a national policy to reduce alcohol and tobacco was implemented (Parliament of the Democratic Socialist Republic of Sri Lanka, 2006). Initiatives included a ban on advertisement and sports sponsorship for alcohol, a minimum age of 21 for sales of alcohol, restriction of alcohol sales on specific religious holidays, increases in prices and taxes of alcohol, and popularisation of lower-strength products assuming they would substitute stronger ones. Recently the Ministry of Finance relaxed laws on alcohol, lifting a ban prohibiting women from buying alcohol, though this was soon reversed (*BBC News*, 2018). In areas known for illicit alcohol consumption and production, numerous government-led raids have been conducted. Small-scale community interventions have proved rather successful in Sri Lanka, including interventions challenging the positive attributes of alcohol (Alcohol and Drug Information Center, 2009) and promoting moderate drinking (Siriwardhana, Dawson, & Abeyasinge, 2012).

Despite these initiatives, alcohol consumption continues to have negative consequences for Sri Lankans, including in relation to poverty (Baklien & Samarasinghe, 2004), domestic violence (Subramaniam & Sivayogan, 2001), depression (Ariyasinghe, Abeysinghe, Siriwardhana, & Dassanayake, 2015), unintentional injuries in children (Punyadasa & Samarakkody, 2016), traffic accidents (Edirisinghe, Kitulwatte, & Senarathne, 2015) and self-harm (Pearson et al., 2014; Sørensen et al., 2017). The rate of cirrhosis is among the highest in the world (Abeyasinghe, 2011).

While a problem-focused understanding of alcohol consumption is important, alcohol is also associated with pleasure and a bearer of cultural and symbolic meanings (Heath, 2007). Drinking is not merely an isolated individual action, but influenced by contextual, social factors structuring behaviour and status (Heath, 2007). Insights into such contextual processes are essential in formulating effective alcohol interventions and policies. Besides from a few in-depth studies (Abeyasinghe, 2002; Baklien & Samarasinghe, 2004; Gamburd, 2008), qualitative research of alcohol patterns in Sri Lanka have been limited.

Other studies have used Bourdieu’s theories to better understand the complex social processes underpinning alcohol consumption, for example, to explore alcohol drinking among adolescence in high-income countries (Järvinen & Gundelach, 2007; Lunnay, Ward, & Borlagdan, 2011). In Järvinen and Gundelach’s mixed-methods study, distinction was utilised to investigate different categories of teenage drinking (Järvinen & Gundelach, 2007). Bourdieu’s concepts have, however, rarely been applied as an analytical framework for research in low- and middle-income contexts.

The aim of this study was to apply Bourdieu’s concepts to explore perceptions and patterns of rural and semi-urban Sri Lankan men’s alcohol consumption practices. Since alcohol consumption is primarily a male activity, we specifically focussed on men’s drinking.

## Methods

### Conceptual framework

To explore individuals’ lived experience, a socio-cultural phenomenological point of view was employed (Creswell, 2013). The aim of exploring perceptions and use of alcohol was inspired by Hunt and Barker’s recommendations for doing socio-cultural drug and alcohol research (Hunt & Barker, 2001). They argue that explorations should view consumption as a social activity, investigating values ascribed to consumption and the setting in which it takes place. To ensure a holistic, contextual exploration of rural and semi-urban Sri Lankan men’s alcohol-related perceptions and behaviour, Bourdieu’s concepts of *practice*, *habitus, symbolic capital* and *distinction* were employed (Bourdieu, 1984).

Bourdieu accentuated that *practice*, in this study ‘alcohol behaviour’, is an interaction between individual decision-making and social structures (Bourdieu, 1977). Though it appears ‘natural’ and consciously learned principles, according to Bourdieu, this logic is deriving from an unconsidered, structural nature of practice (Williams, 1995). This social logic motivating habits derives from the position one has in the social space and is referred to as *habitus* (Bourdieu, 1984)*.* In this study, habitus reflects the social influences and experiences that shape rural Sri Lankan men’s alcohol consumption practices. Alcohol consumption patterns are influenced by the different types of capital available at a certain time. *Social capital* is an individual’s social relations that provide access to resources; *economic capital* is an individual’s revenue and assets; and *cultural capital* is an individual’s social assets such as knowledge acquired through socialisation and education. *Symbolic capital,* the capital primarily expressed in this study, builds on the three other types of capital. It is thus not a specific kind of capital, but every form of capital receiving positive value. Engaging in alcohol consumption thereby helps consumers generate a form of symbolic capital, influencing and communicating one’s position in a certain group (Järvinen & Gundelach, 2007).

Alcohol consumption is not an end in itself, but a way to demonstrate social competencies (Lunnay et al., 2011). Bourdieu explains how individuals engage in certain consumption practices to influence their position, for instance by consuming a specific product or having a certain drinking behaviour (Bourdieu, 1984). Along those lines, Scott et al. used Bourdieu’s concepts to highlight how different forms of alcohol consumption helped construct adolescents’ identity (Scott, Shucksmith, Baker, & Kaner, 2017). Bourdieu furthermore underlines how one group’s alcohol habitus is shaped through *distinction* to the practices of other groups. Certain attributes or drinking patterns become valuable, while others’ patterns are deemed the opposite. For example, a generation can use alcohol to mark distinction to older generations’ practices (Bourdieu, 1984). Using this theoretical frame of *practice*, *habitus, symbolic capital* and *distinction*, alcohol consumption is a symbolic activity influencing rural Sri Lankan men’s social position and self-understanding.

### Study design

Data for this study were collected for 11 months in 2014 and 2015. The qualitative methods focus group discussions (FGDs) and formal and informal observations were employed. The study was nested within a larger qualitative study exploring alcohol’s role in self-harm in rural Sri Lanka, for which in-depth interviews were also carried out (15,29).

### Study setting

This study was conducted in the North Central Province of Sri Lanka, where the majority of the population are Sinhalese, Buddhists. Many families make a living through agriculture or temporary daily wage work and experience irregular income. Although this area is dominated by farming, there are also rapid changes occurring in semi-urban villages. Previous research in this area of Sri Lanka shows high levels of self-harm (Knipe, Padmanathan, Muthuwatta, Metcalfe, & Gunnell, 2017), which have been linked to alcohol consumption as a risk factor (Pearson et al., 2014). Appropriate behaviour is tightly related to norms of morality, respectability and fear of public shame (Obeyesekere, 1984). Obedience towards individuals above one in the hierarchy is crucial for understanding social interactions. This is influenced by gender and age (Marecek, 2006).

### Research team

The research team consists of health and social scientists from Sri Lanka, Australia and Denmark. The first author collected data in collaboration with two experienced Sinhala research assistants who were vital in gaining access, building rapport, translating and explaining observations and social context. A male assistant had substantial experience in participatory research in the area. He negotiated access from village leaders, recruited participants and moderated FGDs. A female assistant had a background in social work and experience in carrying out sensitive interviews. Her main task during FGDs was to interpret to the first author. To overcome challenges met during interpretation, e.g. in regards to sequencing, the team spend time piloting such processes prior to the study. Furthermore, assistants received training about the study and how to carry out FGDs.

### Data

#### Focus group discussions

The primary method of data collection was FGDs, which are well suited when exploring how individuals collectively make sense of a phenomenon (Bryman, 2008). Ten FGDs were conducted with 83 individuals in rural and semi-urban villages with 6–11 individuals in each group. FGDs lasted between 1.5 and 2 hours. Village selection was based on prior good experiences of collaboration with village leaders. Existing literature on alcohol in Sri Lanka (Alcohol and Drug Information Center, 2009; Baklien & Samarasinghe, 2004; Gamburd, 2008; Katulanda et al., 2014; Siriwardhana et al., 2012) and observations indicated different patterns of consumption based on age, gender and location. Participants were therefore sampled and divided into groups based on these patterns (Table 1).

**Table 1.** Overview of focus groups.

	Gender	Location	Participants	Age range and mean age	Occupation
1	Men	Semi-urban	8	18–23 (18.8)	Students working within the service sector
2	Men	Rural	11	19–34 (29.5)	Farming
3	Men	Semi-urban	8	43–58 (52.6)	Farming and daily wage
4	Men	Rural	9	40–67 (56.7)	Farming and daily wage
5	Men	Rural	6	56–72 (62.0)	Farming, daily wage and security forces
6	Women	Semi-urban	6	16–25 (21.0)	Students
7	Women	Rural	9	20–26 (22.0)	Housewives and students
8	Women	Rural	10	30–49 (39.2)	Housewives, farming, daily wage
9	Women	Semi-urban	8	38–62 (48.0)	Housewives, farming, daily wage
10	Women	Rural	8	40–62 (52.0)	Housewives, farming, daily wage

FGDs were held in common village areas or private houses. Two pilot FGDs were conducted to adjust the discussion-guide, which was focused on the significance of consumption, occasions where alcohol should be consumed, preferences in type of alcohol, and perceptions of non-drinkers. FGDs were initiated with an introduction to the study and moderators. With an out-set in the discussion-guide, open discussion and reflection was encouraged, allowing groups to discuss emerging themes. With an outset in the literature and observations, two vignettes of different alcohol patterns and consequences were presented to spur conversation. During FGDs, the researchers noted non-verbal expressions and reactions. Comprehensive notes were made during and immediately after FGDs and were discussed among researchers. All FGDs were audio-recorded, ad verbatim transcribed in Sinhala and translated into English. An external certified Sinhala/English translator quality assured a subset of four interviews. FGDs were conducted until saturation was reached.

#### Observations

To obtain first-hand experience of the topic, discover unusual aspects not mentioned in interviews and explore issues that might be uncomfortable for participants to discuss (Creswell, 2008) observations were carried out. The first author lived in the study area for 11 months, informally observing daily life. When carrying out formal observations to understand how, where and with whom alcohol was consumed, the first author was accompanied by a research assistant. Observations took place in villages at legal and illegal alcohol selling establishments and in households during interviews carried out for the purpose of another study (Sørensen et al., 2017). Observations were also made at social gatherings, such as funerals and weddings. Based on existing literature and FGDs, these settings and occasions appeared representative of where alcohol was consumed. Extensive field notes of alcohol and social behaviour were made during and after observations.

#### Data analysis and management

A deductive thematic analysis was carried out. Transcribed and translated discussions were imported into Nvivo version 11 and read and re-read while coded. In forming thematic patterns, major themes emerged including: norms regarding ‘acceptable’ drinking, social context and space for appropriate consumption; social belonging and distinction from other groups of consumers. Emerging themes were discussed within the study team. Bourdieu’s theoretical concepts were applied to unfold the meanings of the themes.

### Ethical considerations

Ethical approval was obtained from Ethic Review Committee, Faculty of Medicine and Allied Sciences, Rajarata University of Sri Lanka, 2 April 2014 (ERC/2014/014). Participants were introduced to the aim of the study before giving verbal consent to participate. Everyone was advised that no personal information or names should be shared, that participation would be anonymous and that they could withdraw from participation at any time.

## Results

### Three categories of alcohol and substance consumers

Three categories of alcohol consumers emerged, behaving according to each their habitus. They were identified through choice of beverage, quantity of alcohol consumed, time and place of consumption and values ascribed to it. These alcohol consumption groupings included: a group of social, moderate consumers, a group of male abstainers, and a group of heavy alcohol users, referred to as ‘everyday drinking men’ by the study participants.

#### Moderate consumers

The majority of participants explained themselves as belonging to the group of moderate consumers. Their drinking behaviour was a ‘social practice’, where consumption fulfilled important social norms and aspirations. It included two subgroups. The sub-group of traditional, consumers were typically low-income farmers or daily wage workers from rural settings, referred to by participants as middle-aged and older, and dressed in the traditional sarong and dress-shirt. The majority were married and lived in traditional households with a wife, children and often also their own parents. They were punctual in their arrival for the FGDs and discussed the topics in a serious and thorough manner. They mainly consumed alcohol after work in the evenings, with other men in the village.

The other subgroup consisted of a younger generation of men who consumed alcohol as well as other substances. They indicated a modern, experimenting consumption practice compared to the traditional consumers. While all men in this study lived in rural and semi-urban low-income settings, these young men appeared modern, urban and Westernised in their choice of hairstyle and clothing, typically jeans and t-shirts. Most were unmarried, but had girlfriends or fiancés. Especially FGD1 with young men in a semi-urban setting was characterised by a jovial ambience of jokes and laughs. As the traditional consumers, these young men consumed alcohol and other substances with peers in a social setting, however, this occurred both during the day and in the evenings, in covert as well as open social spaces.

#### Abstainers and heavy drinkers

During FGDs and observations, alcohol use was associated with two other habitus: a group of abstainers and a group of men practising heavy and daily alcohol consumption. While these two groups were not well-represented among men in the FGDs, they were often referred to and their behaviour compared to other consumption patterns.

The habitus of the abstainer represented a practice in accordance with perceptions of appropriate Buddhist behaviour, where the aim is to abstain from substances contaminating the mind and body, including meat, tobacco and alcohol (Gombrich & Obeyesekere, 1988).

The men in the habitus of the heavy drinkers were in clear contrast to the group of abstainers. According to all participants, they represented a problematic drinking practice, characterised by daily, extensive consumption in solitude. They displayed an uncontrollable, embarrassing behaviour in public, creating problems for others, for example by using an unacceptable language and displaying a violent anti-social behaviour.

### The symbolic capital in choice of alcohol

Symbolic capital appeared to be connected to specific types of alcohol. A clear hierarchy of beverages existed with status enhancing or diminishing values ascribed. For the traditional consumers, the choice of alcohol was cheap products with a high alcohol volume. For them, the most commonly consumed alcohol products were therefore kasippu and arrack. The cheap kasippu, an illegal homebrew made from fermented fruits, was typically sold in small, blue plastic bags in villages. To avoid police raids, sellers had established different measures to secretly reach their customers, for example by hiding a batch of kasippu near a lake, informing customers where to fetch it. Interestingly, in this male-dominated field of alcohol consumption, the majority of sellers were women. Arrack, a legal spirit distilled from fermented sap of the coconut palm, was sold in bottles in liquor stores in close proximity to villages.

The choice of alcohol was influenced by the economic capital available to men. With a high alcohol volume, kasippu was known as good value for money and older men in a semi-urban setting mentioned how they preferred kasippu out of ‘necessity’ due to the lower price tag. When they could afford it, they consumed larger amounts, preferably of the slightly more expensive arrack. In periods of low income they would revert to the cheap and easily available kasippu. In contrast, the young, experimenting consumers emphasised how they would never choose kasippu, no matter their financial status:

Elders are used to drinking kasippu– it is easy and close by. Young people would rather go a little far and obtain good stuff instead. (FGD 2)

This ‘good stuff’ was either beer, which they perceived as being ‘ok to drink and good for the body too’, or imported spirits. Local liquor stores sold international and Sri Lankan brands of beer as well as imported Western brands of strong spirits. For a larger selection of brands and up-scale spirits and wines, villagers frequented shops in larger cities. A general notion was that more costly products carried more prominence.

Positioning themselves in the community as having a certain level of prestige, the young, experimenting men’s drinking preferences appeared to be a marker of symbolic capital, communicating a modern lifestyle in opposition to the farming lifestyles they were brought up in. The younger men expressed kasippu to be a marker of low social status, having an ‘ugly’ name and kasippu-consumers as ‘smelly’:

There is normally too much odour when you get close to someone who drank kasippu. When you walk close by that odour comes out. When you smell it you know it is Kasippu odour. (FGD10)

The young men made a distinction to kasippu-drinkers who they saw as equivalent to heavy drinkers, representing low morality and excessive drinking. In explaining this distinction from their own consumption towards older, traditional consumers’, the younger men emphasised how they experimented with a range of non-alcoholic substances. This included non-prescription painkillers, cough syrup, cannabis-tobacco mix and ayurvedic medicine, which they obtained from pharmaceutical outlets, ayurvedic shops or from bus stations, where illegal substances were distributed from other places in the country. The young men preferred these other substances, partly due to their status-enhancing attributes:

There is one called Dunhill. That is like a cool cigarette. (…) That one is cool and that is like when you eat a toffee [cannabis mixed substance in form of a toffee]. [You] feel cool like that. (FGD 1)

The traditional, often older, consumers disassociated themselves from the younger generations’ consuming habitus. They thought of the youth as having a pretentious lifestyle and consumption pattern, while also enviously describing them as spoiled, having better opportunities than they ever had:

Generally youth in this area here (…) have work. When it is the season for chillies they pluck chillies. That is some kind of work. When they do that, they collect money. Then without the permission of the parents they go and buy [alcohol]. No. When they want something they do it: Go to the bar and drink arrack, buy phones, watch films and do various things. (…) parents don’t even know about it. (FGD5)

These men were thus not only estranged to the products consumed by the younger men but to their lifestyle and opportunities available to them. The choice of consumption enhanced the message of a modern lifestyle, in the view of themselves but also the older generation.

### Practicing social belonging through appropriate consumption

With habitual variations, the older and younger participants in this study shared an understanding of acceptable alcohol consumption and navigated within three areas of alcohol behaviour: to consume alcohol publicly or covertly, in a social setting or alone and in moderate or excessive amounts.

#### Public or covert consumption

All participants expressed how alcohol consumption should take place in hiding. Besides from social occasions, alcohol drinking should never occur in public or in view of non-drinkers. Further, it was atypical for men from different age groups to consume alcohol together, for example, a father and son. Observations confirmed that it was uncommon to see formal, public places where alcohol could be enjoyed. However, small plastic bags scattered throughout some villages revealed that kasippu was consumed. In FGDs it was explained how kasippu was quickly swallowed after tearing the plastic bag, so that the consumer could drink it unnoticed. Empty beer-cans found by the edge of lakes and paddy fields also bore witness to how men consumed alcohol there ‘in private’. All men emphasised how they would never bring alcohol to their household. Older men in a rural setting explained:

That means, now we two had a talk to drink it secretly, then people at home don’t know and also the community doesn’t know. We do not drink in front of the people at home. That [alcohol] is brought somewhere and drunk in hiding. Otherwise the wife will scold you all the time. Nobody drinks openly. (FDG 4)

Similarly, young men highlighted how gatherings involving alcohol would take place in hiding ‘where messages don’t go to anyone at home’. If a party *had* to be held at home, the young men chose substances without smell, absorbed orally over time – such as cannabis-induced tobacco – to hide consumption from non-drinkers and older generations.

Public drinking was in FGDs explained to be associated with excessive consumption and a troublesome behaviour, affecting bystanders and family members. This included violent fights with spouses and a difficult study-environment for children. It was underlined that a man consuming alcohol in public failed to live up to the norms of appropriate behaviour, thereby exhibiting poor moral judgment. In the FGDs, all men referred to public drinking as distanced from their own behaviour, but connected to the habitus of the heavy drinkers and their uncontrollable consumption patterns.

#### Social or solitary consumption

Although covert drinking was widely practiced, solitary consumption was inappropriate. Both genders and all age groups linked solitary drinking to the habitus of the heavy drinkers. Instead, alcohol should be consumed with others in a social setting:

If I got the thought that now I would like a drink, I would not do it. Generally two or three [men] get together and drink. At least you will be joined by another person. (FGD 2)

Value was given to the social ties and connections gained from drinking and consuming ‘for fun’ in social groups was mentioned by all men. To serve and consume alcohol was anticipated in certain settings, for example at key social Sinhalese rituals, such as weddings and New Year celebrations. Rural, middle-aged women explained:

When they have a party, alcohol is everywhere. If we do not give alcohol to our party, it will feel like we left something out. (FGD 8)

Hence, symbolic value and social recognition was attached to ‘being able to serve’ abundant amounts of expensive spirits at key social events, thereby communicating social status.

At such occasions, men consumed alcohol in an entirely male sphere. The habitus of consumption was age-specific and men assembled with others from their own age-group. Wedding observations showed that while men of all ages got increasingly drunk, louder and dancing, women disappeared to quiet areas. Mid-aged women from a semi-urban area mentioned how:

They [the men] go to a side, so we just get dressed well (…) So we just sit on a chair and wait, then eat and go home. [We] have fights, so feel ashamed to come out from the home on the following day. We have felt the loneliness. (FGD 9)

Though consumption was expected by both gender, the women thus articulated how they did not approve of it.

#### Timing of consumption

When there was no social occasion to attend, all participants in FGDs explained how a rule of proper consumption was to only consume alcohol after dark in a social setting. For many men, consumption took place in an almost ritualised form nearly every night:

Normally, it is in the evening. There is a normal drinking time in the evening. After it has passed seven. (…) after it is dark, everybody starts drinking [alcohol]. (FGD 4)

Older and middle-aged alcohol users explained how the nightly alcohol drinking was a central way of socialising, especially after a day of work in the fields. When assisting others with farming practices it was expected to pay them in alcohol.

The young, experimenting men viewed nightly drinking as appropriate for alcohol, but had different standards for consuming other substances. Developing their own social codes of consumption, these substances were consumed during the day, even in front of individuals traditionally perceived to be above them in the hierarchy. While the young men explained it to enhance educational performances it also had a social component, and it was explained to be obvious to peers: ‘If we go to class and have taken a pill, we laugh, it must be shown to the others’. The young men thereby not only distinguished themselves from older consumers through choice of product, but also through timing and setting in which it was consumed.

### Navigating between abstainers and heavy drinkers

The amount consumed was important for men to stay within norms of acceptable alcohol behaviour. The moderate consumers generally viewed their alcohol intake as controlled and limited to social settings. The subgroup of young, experimenting men, however, saw their own practices in contrast to the older generation, describing *all* older men as heavy drinkers. Distancing themselves from the behaviour of the heavy drinkers, older and middle-aged men justified *their* drinking at night as a means to relief stress after a day of hard work without causing trouble.

So in the evening at the time of having dinner, [I] just have a bath and take a drink and then go to sleep. So this is not like a person who has been drinking alcohol since the morning, who shouts and does this and that. (FDG 5)

While there was a clear distinction made towards the heavy drinkers, the subgroups of moderate consumers did not make congruent distinctions towards abstainers. For the older and middle-aged traditional alcohol consumers, abstaining from drinking alcohol displayed strong symbolic capital, which abstainers were commended for. Several older men sought to position themselves as such, highlighting how they had limited their alcohol intake, aspiring towards high moral standards and better health.

Conversely, among the younger alcohol consuming men, abstainers were perceived as outsiders, mentioning how some adolescents did not drink and were thereby excluded from their social group:

There are some, who are like flowers. They are kept in the home and not allowed to associate [with the] community. (…). They associate very little with us in the sense that they do not go anywhere at night, they are not allowed to come out. (FGD 1)

The assumption that non-drinkers were fragile was noted through their description as ‘flowers’.

The group of moderate drinkers thus positioned themselves differently on the spectre of consumption. For the older and middle-aged traditional consumers, abstaining from alcohol was behaviour to pursue, communicating purity and aspirations towards a better self, while making a clear distinction to the heavy drinkers. The younger men, on the other hand, positioned themselves in between the socially dissociated abstainers and the inappropriate behaviour of the heavy drinkers.

## Discussion

This study investigating alcohol consumption in rural and semi-urban Sri Lanka brings forward several contributions to the field of alcohol research. To the authors’ best knowledge, this is the first study using Bourdieu’s concepts to analyse alcohol consumption in a middle-income country. A web of factors influenced the participants’ consumption patterns. Three categories of consumers emerged, highlighting how rural and semi-urban Sri Lankans of different generations are not a homogenous group of consumers. Especially the portrayal of the young experimenting consumers is new in this context of Sri Lanka. Bourdieu’s concepts helped to highlight interesting tensions between the different habitus of consumers, including how there were (i) expectations of providing alcohol at Sinhalese rituals, (ii) how social connections were forged in drinking culture and (iii) how young men embraced a range of substances. All of these ran counter to Buddhist ideals and traditional expectations to consumption.

### Young men’s consumption lifestyle

The alcohol consumption patterns exposed in this community, appears reflective of larger societal structures and changes in Sri Lanka – a country experiencing socio-economic growth (World Bank, 2017). It seems that countries with economies undergoing rapid transition are increasingly affected by different types of drug use (United Nations Office on Drugs and Crime, 2012). An Indian study showed that societal changes influenced opportunities and financial resources available to adolescence and thus their consumption patterns (Chowdhury, Ramakrishna, Chakraborty, & Weiss, 2006). In our study, a distinction between the generations through substances used and time and place of consumption was highlighted. This appeared emblematic of how the young, experimenting men generally lived their lives, reflecting a different lifestyle.

The consumption patterns associated with the habitus of ‘abstainers’ and ‘heavy drinkers’ were brought forward to communicate distinction and belonging to social groups. This is a typical Bourdieu taxonomic struggle between ethics of sobriety and indulgence, restraint and fun (Bourdieu, 1984). Similar to findings from a Western context (Järvinen & Gundelach, 2007), the young men in this study saw abstaining male peers as lacking social capital and thus excluded from social belonging. In a context that has traditionally praised abstainers this is a significant shift and a clear distinction to the traditional consumers. While other studies in Sri Lanka identified the same feminine accounts of abstainers, it was in that context perceived as a positive value (Gamburd, 2008).

Youth alcohol consumption has been investigated in a few studies in Sri Lanka (Perera & Torabi, 2009) and the use of other substances was explored by Liyanage et al. in a study among urban school children in the capital of Sri Lanka (Liyanage et al., 2013). The latter study found that urban male adolescents consumed the same substances as those identified in the present study. Experimenting substance use is thus no longer limited to an urban setting, but also an activity among young men in semi-urban settings of Sri Lanka.

### The social component of alcohol consumption

It appeared important for both subgroups of moderate consumers to classify their consumption as moderate, socially acceptable and appropriate. Along the lines of MacAndrew and Edgerton’s argument that ‘drunken comportment’ is not universal but culturally constructed (MacAndrew & Edgerton, 2003), the behaviours highlighted in this study should be seen within the context of Sinhalese values, where dramatised expressions of emotions are discouraged and modesty and restraint valued (Marecek, 2006; Obeyesekere, 1984). As Gamburd pointed to in her ethnography of illicit alcohol in southern Sri Lanka, perceptions of problematic drunkenness implied to drink at times perceived as ‘inappropriate’ and loss of self-control (Gamburd, 2008). This was evident in this study, where consequences of uncontrolled consumption behaviour entailed breaking the social code of appropriate conduct. MacAndrew and Edgerton argue that drunken comportment provides a ‘time-out’ from one’s usual behaviour, though always within limits (MacAndrew & Edgerton, 2003). In a previous study, we argued that inebriation seemed to provide rural Sri Lankan men with a ‘time-out period’, where otherwise inappropriate behaviour was tolerated (15).

In line with Buddhist and Sinhala values, and due to the many harmful socio-economic and health consequences of alcohol (Baklien & Samarasinghe, 2004; Sørensen et al., 2017), reduction of alcohol and drug consumption is high on the political agenda in Sri Lanka. The stated aim of the Government is to gradually eliminate alcohol, drug and tobacco use through national polices (Ministry of Health, Nutrition and Indigenous Medicine, Sri Lanka, 2016) and the alcohol industry has been labelled a ‘sin industry’ (Dayaratne, 2011). As Järvinen pointed out, codes for legitimate alcohol drinking are likely to affect both the way people consume but also how they verbalise it (Järvinen, 2001). When the men described their consumption as ‘appropriate’, this may reflect a desire to appear responsible, adjusting their behaviour to the health recommendations put forward in the country. What we have presented in this study might therefore not only be representations of practice, but also presentation of a version of an ideal self (Järvinen, 2001).

The identified social practices may be useful in informing future alcohol and health research and promotion strategies on how different consumers might be understood. It should be noted that though the abolishment of alcohol consumption is high on the agenda, it is complicated by several aspects. This study highlights how the availability of financial means influence choice of consumption and policies targeting taxation on legal alcohol is likely to not only impact on the trade of illicit alcohol, in particular in rural areas (Dayaratne, 2013), but potentially also drive young consumers to riskier behaviour using uncontrolled substances.

## Limitations

While this study sought to have a holistic approach to exploring alcohol consumption in rural and semi-urban Sri Lanka, a few limitations should be noted. Several relevant groups had low representation in the FGDs, including abstainers, heavy drinkers and older, semi-urban consumers (above the age of 62). This is likely to have influenced the findings.

Bourdieu’s concepts brought a useful frame to explore alcohol consumption. Especially Bourdieu’s concept of ‘habitus’, however, has also been criticised for not considering the individual’s ability to consciously change their life situations (Yang, 2014). Although this study aimed to include the wider context, the framework utilised might not have captured variations in practice and potential slippage between the categories of consumers portrayed. Tension within the different habitus was found, including important outliers such as young men who did not consume other substances than alcohol and older men who did not consume every night with other men in the village. That said, Bourdieu’s concepts presented a helpful framework to capture the social structures at play in alcohol consumption in rural and semi-urban Sri Lanka.

## Conclusion

This study explores alcohol consumption among rural and semi-urban Sri Lankan men, contributing with an in-depth understanding of alcohol consumption as a social phenomenon, mirroring changing social structures. This study identified three groups of consumers who distinguished themselves from others through their consumption. Appropriate consumption was defined as ‘moderate consumption’ in covert, social and contained settings. Symbolic capital was expressed through choice of alcohol. The findings highlight how rural and semi-urban Sri Lankan alcohol consumers do not belong to one homogenous group and how consumption is an important social activity among certain groups of men. The characteristics of each group of consumers should be considered when designing policies and programmes seeking to decrease the harmful effects from alcohol.
